# Early Life History Divergence Mediates Elevational Adaptation in a Perennial Alpine Plant

**DOI:** 10.1002/ece3.70454

**Published:** 2024-10-21

**Authors:** Aksel Pålsson, Ursina Walther, Simone Fior, Alex Widmer

**Affiliations:** ^1^ Institute of Integrative Biology ETH Zurich Zurich Switzerland

**Keywords:** fitness trade‐offs, life table response experiment, matrix population models, natural selection, phenotypic plasticity, reciprocal transplant experiment

## Abstract

Spatially divergent natural selection can drive adaptation to contrasting environments and thus the evolution of ecotypes. In perennial plants, selection shapes life history traits by acting on subsequent life stages, each contributing to fitness. While evidence of adaptation in perennial plants is common, the expression of life history traits is rarely characterized, limiting our understanding of their role in adaptive evolution. We conducted a multi‐year reciprocal transplant experiment with seedlings from low and high elevation populations of the alpine carnation *Dianthus carthusianorum* to test for adaptation linked to contrasting climates and inferred specific contributions of early life stages to fitness. We assessed genotype by environment interactions in single fitness components, applied matrix population models to achieve an integrated estimate of fitness through population growth rates, and performed trade‐off analyses to investigate the advantage of alternate life history traits across environments. We found evidence of genotype by environment interactions consistent with elevational adaptation at multiple stages of the early life cycle. Estimates of population growth rates corroborated a strong advantage of the local genotype. Early reproduction and survival are alternate major contributors to adaptation at low and high elevation, respectively, and are linked by trade‐offs that underlie the evolution of divergent life history traits across environments. While these traits have a strong genetic basis, foreign populations express co‐gradient plasticity, reflecting the adaptive strategy of the local populations. Our study reveals that selection associated to climate has driven the evolution of divergent life histories and the formation of elevational ecotypes. While the high energy environment and strong competition favor investment in early reproduction at low elevation, limiting resources favor a more conservative strategy relying on self‐maintenance at high elevation. The co‐gradient plasticity expressed by high‐elevation populations may facilitate their persistence under warming climatic conditions.

## Graphical abstract

In perennial plants, natural selection shapes life‐history traits during subsequent stages of the life cycle, but despite the close link of such traits to fitness, their adaptive role is rarely characterized. Using a multi‐year reciprocal transplant experiment, we test for adaptation of the alpine carnation *Dianthus carthusianorum* in response to different climate regimes at contrasting elevations in the central European Alps, and assess the contribution of early life‐history traits through integrative analyses of demographic models and fitness trade‐offs. We find that elevational ecotypes are characterized by alternate life‐history strategies that underly adaptation, but also show high levels of plasticity in response to climatic variation: while a more conservative strategy securing self‐maintenance is beneficial at high elevation, a strategy with high investment in reproduction is beneficial at low elevation.

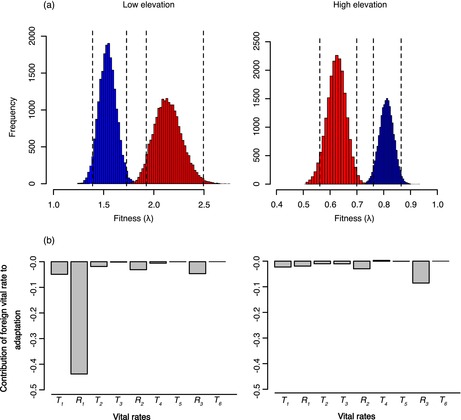

## 1 Introduction

Spatially divergent natural selection drives adaptation of populations to contrasting environmental conditions (Savolainen, Lascoux, and Merilä 2013) and may lead to the evolution of phenotypically divergent ecotypes (Turesson 1922; Wadgymar et al. 2022). Adaptation in plants is well studied owing to their amenability to reciprocal transplant experiments, where fitness, i.e., the contribution of an individual or genotype to the next generation, can be quantified in different environments. In annual plants, fitness is commonly estimated through seed number, as this captures life‐time reproductive success (Ågren and Schemske 2012; Bischoff and Hurault 2013). In perennial plants, the matter is more complicated, because reproduction depends on selection acting on successive stages of a multi‐year life cycle, with each stage making a complementary contribution to life‐time fitness, and the inherent costs of each life stage may imply trade‐offs with others (Kim and Donohue 2011; DeMarche, Anger, and Kay 2020; Wadgymar, Daws, and Anderson 2017). As a result, populations growing in different environments may evolve life history traits that optimize the allocation of resources at different stages of the life cycle to maximize life‐time fitness (Stearns 1992; Friedman 2020; Forbis and Doak 2004; Shefferson et al. 2003). To understand adaptation in perennial plants, it is thus essential to quantify selection across multiple stages of the life cycle and dissect how interactions between fitness components shape the evolution of divergent life histories.

Life history traits of perennial plants, such as age‐ or size‐specific reproduction and longevity, characterize the investment of individuals in different fitness components (Stearns 1992; Laiolo and Obeso 2017; Acasuso‐Rivero et al. 2019). Life history theory predicts interactions between the expression of individual fitness components that depend on resource allocation (Stearns 1992; Friedman 2020; Hamann, Wadgymar, and Anderson 2021).

In resource‐rich environments where nutrient availability or climatic conditions do not impose strong selection pressure, investments in one fitness component can have positive effects on others (e.g., Biere 1995; Villellas and Garcia 2017). In most natural environments, however, resources are limited, and their allocation is typically associated with trade‐offs. A classic example is investment in reproduction, which can lead to reduced future survival and growth (Obeso 2002; Sletvold and Ågren 2015; Hamann, Wadgymar, and Anderson 2021). In general, trade‐offs can vary across the life span of perennial plants and can be exacerbated under adverse environmental conditions (Stearns 1992; Acerenza 2016; Hamann, Wadgymar, and Anderson 2021; von Euler, Ågren, and Ehrlén 2012). Hence, contrasting selection pressures in different environments can lead to the evolution of ecotypes expressing divergent life history strategies (Stearns 1992; Childs, Metcalf, and Rees 2010; Friedman 2020; Boyko et al. 2023). Characterizing the adaptive value of life history traits requires field experiments where fitness is modeled in an integrative framework to assess stage‐specific contributions. Yet, despite the importance of life history traits in the evolution of perennial plants, this is rarely done.

Matrix population models (MPM) offer a powerful analytical framework to estimate population growth rates by integrating fitness and trade‐offs across multiple fitness components of the life cycle (Caswell 2001). When applied to multi‐year reciprocal transplant experiments, MPM provide an integrative estimate of fitness suitable to compare the performance of populations. Life‐table response experiments (LTRE) are analytical methods applied to MPM that can identify vital rates, i.e., stage or age‐specific fitness components, with the strongest contributions to adaptation (Caswell 1989). The influence of specific vital rates on population growth is expressed as elasticities. Age classes that compose each population are estimated as stable age distributions (Caswell 2001). Together, these estimates can provide complementary descriptors of the impact of different life history traits on the responses of populations to different environments. Even though MPM are a powerful tool for life history analyses, they remain underutilized in adaptation studies (Wadgymar et al. 2022; but see e.g., Waser and Price 1985; DeMarche, Kay, and Angert 2016; Goebl et al. 2022), because they require comprehensive datasets that ideally include seedling establishment, a stage of critical importance for plant fitness but relatively rarely assessed in the field (Kitajima and Fenner 2000; Kim and Donohue 2011).

Understanding the contributions of life history traits to fitness is particularly important for species that are predicted to face substantial environmental change, such as alpine plants (Anderson and Song 2020; Tito, Vasconcelos, and Freely 2020; Lancaster, Morrison, and Fitt 2016). Plant populations growing along elevational gradients typically experience contrasting environmental conditions over short geographic distance, and often show evidence of local adaptation (Halbritter et al. 2018). While alpine plants are typically long‐lived species that are challenging to survey in transplant experiments, early stages of their life cycles are crucial for establishment and start of reproduction, thus assessing their role in adaptation is key to understand the evolution of distinct life history strategies. Plant life history traits often vary along elevational gradients, which suggests that different life history strategies may be advantageous under contrasting environmental conditions (Laiolo and Obeso 2017). While such variation may have a genetic basis, it may also result from phenotypic plasticity, the ability of genotypes to express different phenotypes in different environments (Price, Qvarnström, and Irwin 2003; Ghalambor et al. 2007; Acasuso‐Rivero et al. 2019). Considerable plasticity has been documented in life history traits across a wide range of organisms (Davidson, Jennions, and Nicotra 2011; Palacio‐Lopez et al. 2015; Ensing and Eckert 2019), and while there is no consensus on the extent to which plasticity influences adaptation, plasticity that is expressed in response to changing conditions can potentially support transient population persistence (Fox et al. 2019; Nicotra et al. 2010; Matesanz, Gianoli, and Valladares 2010).

Here, we assess the contributions of early life history traits to elevational adaptation linked to climate variation in the perennial alpine carnation *Dianthus carthusianorum* through a multi‐year reciprocal transplant experiment of young seedlings across elevation in the Alps. We recorded individual fitness components through multiple stages of the plant life cycle spanning three flowering transitions, and integrated empirical estimates of establishment to estimate population growth rates. We test for elevational adaptation through evidence of genotype by environment (GxE) interactions driven by natural selection and dissect the contribution of life history traits to this process through analyses of life table responses experiments and fitness trade‐offs. Using this experimental framework, we ask: (1) Are populations of *D. carthusianorum* adapted to their elevation of origin? (2) Which early stages of the life cycle contribute to adaptation, and are fitness components linked by trade‐offs that underly the evolution of divergent life history traits? (3) Is the expression of early life history traits plastic and if so, do populations growing in a foreign habitat express co‐gradient plasticity?

## 2 Materials And Methods

### 2.1 Study System

*Dianthus carthusianorum* L. (Caryophyllaceae) grows on dry and nutrient poor grasslands and rocky slopes at elevations from sea level up to the alpine zone above 2100 m asl in the European Alps (Landolt 1985; GBIF 2022). It is a short‐lived herbaceous perennial with a woody taproot and a basal rosette of grass‐like leaves. Upon flowering, it produces one to several mostly unramified generative stalks, each with a single terminal inflorescence consisting of up to 15 pink to purple flowers (Garcke 1972). *D. carthusianorum* is gynodioecious, self‐compatible but primarily outcrossing, and main pollinators are diurnal butterflies (Bloch, Werdenberg, and Erhardt 2006).

**FIGURE 1:**
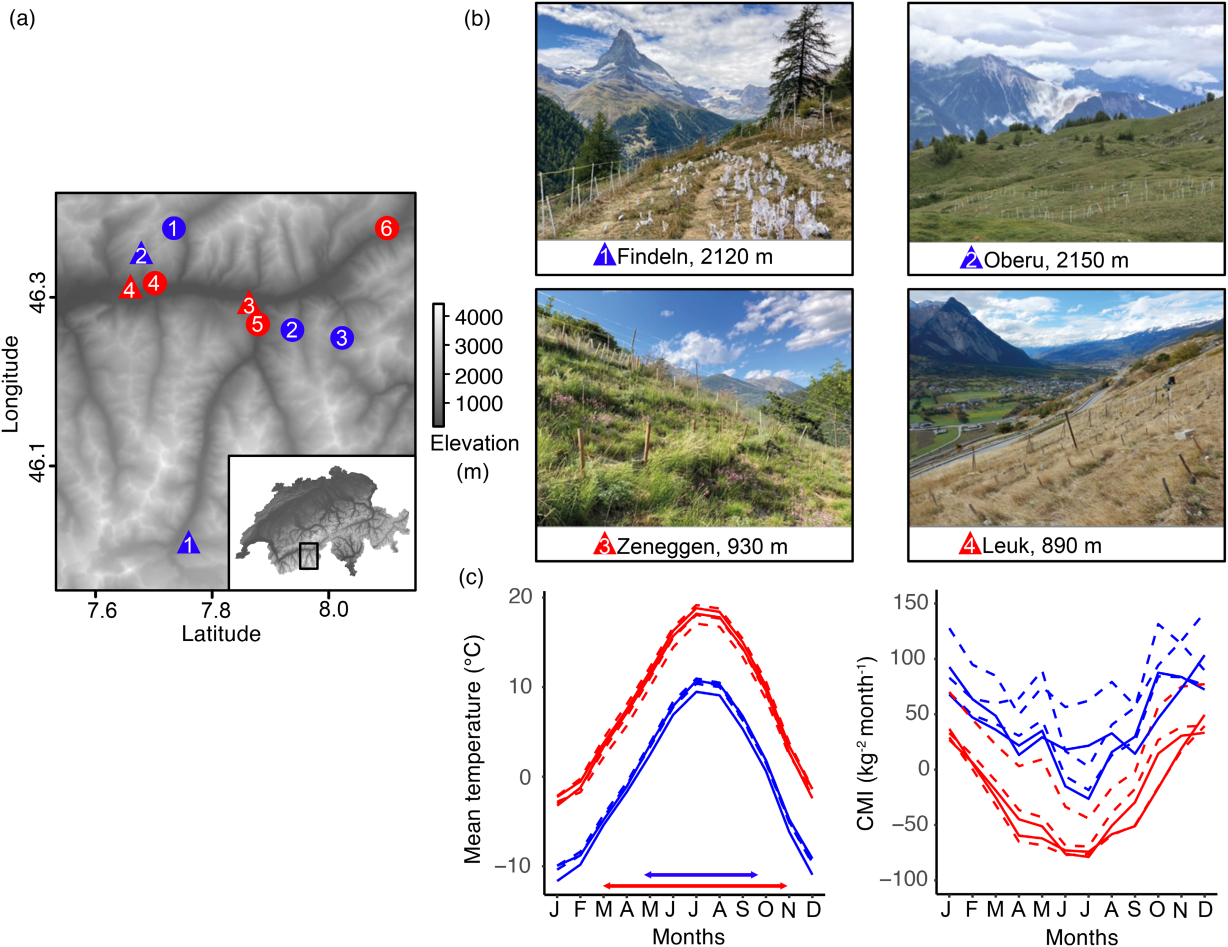
Experimental set up of reciprocal transplant experiments. (a) Locations of the sampled *D. carthusianorum* populations (circles) and the transplant sites (triangles) in the study area in the Upper Rhône Valley (Switzerland), red and blue denote low and high elevation, respectively. Numbers follow Tables S1 and S2. (b) Low (bottom panels) and high elevation (top panels) transplant sites. (c) Mean monthly temperature and climate moisture index (CMI) at low (red) and high (blue) elevation transplant sites (filled lines) and at the original sites of the wild populations (dashed lines). CMI indicates the difference between amount of precipitation and potential evapotranspiration, negative values are hence associated with dry conditions. Estimates are the monthly averages calculated over the timespan 1980–2018 using the Chelsa high‐resolution data base (Karger et al. 2021). The arrows indicate the growing season.

Our study was performed in the in the Upper Rhône Valley (Valais, Switzerland) in the central Alps (Figure 1), a major east–west oriented inner‐Alpine valley with a dense yearly precipitation distribution leading to a particularly dry climate at low elevation (Braun‐Blanquet 1961; Zumbrunnen et al. 2009). Erosion during Pleistocene glaciations generated a high relief landscape with peaks over 4000 m (Sternai et al. 2013), resulting in pronounced climatic gradients between dry and warm conditions at low elevation, and wetter and cooler conditions at higher elevation. In September 2015, we established two common gardens each at low and high elevation (~900 and 2100 m, respectively; Table S1). We established a reciprocal transplant experiment with populations originating from opposite ends of the elevational distribution range of *D. carthusianorum* in the Upper Rhône Valley. Three low (i.e., ~700–900 m) and three high (i.e., ca. ~2000–2200 m) elevation populations were sampled in neighboring valleys to represent the species' distribution in the colline (i.e., lower elevation vegetation zone < 900 m;) and alpine (i.e., above timberline) elevational belts (Rubel et al. 2017; Table S2). Population genetic analyses have shown recurrent gene flow between populations across the elevational gradient (SF, unpublished results). Each transplant site was equipped with a weather station (DS3 IP66, SensorScope, Lausanne, Switzerland). Comparison of climate parameters with long term records (i.e., 1980–2018) from the Chelsa database (Karger et al. 2017, 2021) indicate that the transplant sites capture the different climate experienced by the six populations at their original sites (Figure 1C; Figures S1 and S2). Soil potential measured at a depth of 30 cm further highlights the summer drought that affects the low elevation sites (Figure S3).

### 2.2 Reciprocal Transplant Experiment

We collected seeds from 20 to 39 plants (i.e., maternal families) in each of the six focal populations in fall 2012 and 2014. In summer 2015, we germinated the seeds and grew seedlings over a period of 3 months in a greenhouse in peat moss‐based soil (Klasmann Deilmann Gmbh) under a 12‐h day/night cycle, with temperatures set to 20°C and 18°C during the day and night, respectively, and a relative humidity of 50%–60%. In fall 2015, we transplanted ~500 seedlings from 127 to 135 maternal families at each of four transplant sites (Tables S1 and S2). Seedlings were placed in eight randomized blocks, approximately 1 m apart. Each block contained 72 individuals, arranged in a 12 × 6 grid and spaced 25 cm apart. As the start of winter following the onset of the experiment was exceptionally warm and dry, we watered seedlings twice a week until mid‐January to help establishment. Individuals that died within 2 weeks after being transplanted were attributed to transplant shock and excluded from the analyses. The sites were fenced to exclude large herbivores commonly present in the study area, such as deer, cattle, and marmots. Because our focus is adaptation to the abiotic environment, we limited the influence of above ground competition with neighboring plants by regularly trimming the surrounding vegetation.

### 2.3 Assessment of Vital Rates

We monitored vital rates, i.e., age specific survival and reproduction, over the growing seasons 2016–2018, encompassing three reproductive transitions. Because these vital rates represent the plants' relative investment in fitness components across different stages of the life cycle, they characterize the life history traits of our elevational genotypes. At the low sites, we defined the start and end of each growing season as the first and last date of 6‐day windows with mean daily temperatures above 10°C. Because the weather stations had to be removed during winter at the high sites to prevent damage from snow, we defined the start of the growing season based on the observed start of vegetative growth, which approximately corresponded to 3 weeks after snowmelt. The growing seasons ranged from March to November at low elevation, with flowering occurring typically in June, and from late May to October at high elevation, with flowering primarily in July (Figure 1). We recorded survival at the start and end of each growing season and visited each site twice a week in 2016 and once a week in 2017 and 2018 to record flowering individuals. We bagged individual inflorescences in synthetic mesh bags for seed collection after flowers had wilted. In the laboratory, we extracted seeds from capsules by carefully separating undeveloped seeds and quantified seed number as the average number of mature seeds from two independent runs of an elmor C3 High Sensitive Seed Counter (Elmor Ltd., Schwyz, Switzerland). We estimated the size of the basal rosette at the start and end of each growing season (except the end of the second year) from high resolution pictures of each plant (Nikon D810; 7360 × 4912 pixels) including a reference standard, and by computing the area based on two orthogonal diameters measured in image J v.2.0 (Schindelin et al. 2012). This estimate of plant size provides a suitable proxy for biomass as evidenced by its correlation with plant dry weight (Figure S4).

### 2.4 Seedling Establishment

We assessed germination and seedling establishment success of all six focal populations growing in each transplant site for implementation in MPM. From the seed harvested in 2017 at each site, we selected ~200 seeds from each population represented by fruiting plants belonging to up to five maternal families originally collected in the wild populations. At the high elevation sites, one of the low elevation populations could not be included in the experiment due to low seed production. To obtain establishment specific of each transplant site, in fall 2018 we sowed ~10 seeds per maternal family in the same sites in which they were produced, using peat moss soil (Klasmann Deilmann Gmbh) contained in biodegradable pots of 10 × 8 cm. We placed 96–165 pots per site in the ground after random assignment to positions within the blocks of the original experiment left empty by dead individuals. Plants that germinated successfully and were alive at the end of the 2019 growing period were considered established.

### 2.5 Statistical Analyses

#### 2.5.1 Individual Vital Rates

We assessed elevational adaptation from individual vital rates including flowering probability, seed number and survival, and tested for differential plant size. We tested for genotype by environment interactions, as well as for differences between genotypes growing within each environment (local vs. foreign criterion for local adaptation) and for the effect of the environment on each genotype (home vs. away criterion, Kawecki and Ebert 2004). We fitted individual generalized linear mixed effect models for flowering probability, seed number and survival at subsequent stages of the life cycle (Table S3). In each model, we implemented the vital rate as response variable and genotype, transplant environment and their interaction as predictors, using a binomial error distribution for the categorical variables (i.e., flowering probability and survival), and a zero‐inflated Poisson error distribution for the count variable (i.e., seed number). We analyzed plant size using the same model structure as above, but implemented in linear mixed effect models with a Gaussian error distribution and we log transformed the data to improve distribution of the residuals. In all analyses, we implemented maternal family nested within population and block nested within site as random effects. We implemented mixed effect and zero‐inflated models using the R package lme4 v1.1 (Bates et al. 2015) and glmmTMB v. 1.3 (Brooks et al. 2017), respectively, and used DHARMa v. 0.4.6 to perform model diagnostics of the zero‐inflated models (Hartig 2022). We assessed significance levels of interactions using likelihood ratio tests. We obtained estimates, significance levels and confidence intervals of the contrasts between genotypes with the emmeans package (Lenth 2017). We analyzed survival throughout the life cycle using mixed effect cox models, which perform proportional hazards regression of time to event data with implementation of random effects. We fitted the Cox models with genotype and transplant environment and their interaction as predictors and used the same random effect structure as used in the mixed effect models in the package survival 2.44 (Therneau and Grambsch 2000). All analyses were performed in R v.3.3.2 (R Core Team 2022).

#### 2.5.2 Integrated Fitness Estimate

We formulated age‐structured MPM to obtain an integrated estimate of fitness expressed as population growth rate λ for each genotype growing at low and high elevation. To obtain survival and reproductive vital rates, we divided the plant life cycle into winter (W_i_) and summer (S_i_) stages (Figure S5). We calculated the survival vital rates as the proportion of individuals transitioning (T_i_) to the next life stage. To achieve a comprehensive estimate of reproduction, we used an integrated value of the reproductive vital rates (R_i_) and used the product of flowering probability, seed number and recruitment. From the seedling experiment, we estimated the establishment rates per genotype in each site from generalized linear mixed effect models by implementing the proportion of seedlings established per pot as the response variable and genotype as the predictors, with binomial error distribution and the same random effects structure as described above for the single vital rate analyses. We recovered similar establishment rates of the two genotypes conditional to the growing environment, with overall higher values in the low (i.e., low genotype: 0.112 ± 0.041; high genotype: 0.098 ± 0.038; Table S4) compared to the high elevation environment (i.e., low genotype: 0.033 ± 0.125; high genotype: 0.051 ± 0.021; Table S4). To assess whether differences in population growth rates between the genotypes growing in each environment are statistically significant, we performed 20,000 bootstrap replicates of each matrix stratified by population, and constructed bias corrected 95% confidence intervals around estimates. To decompose the effects of specific vital rates on λ, we used life‐table response experiments (LTRE) (Caswell 1989). LTREs tests the difference in λ between a matrix of interest and a reference matrix, breaking down the contributions of subsequent vital rates of alternate genotypes. We compared the matrix of the foreign genotype against the matrix of the local genotype in each environment. We consider the inferred differences to identify alternate life history traits that contribute to elevational adaptation. All MPM analyses were performed using packages popbio v. 2.2.4 (Stubben and Milligan 2007) and boot v. 1.3 (Canty and Ripley 2021).

#### 2.5.3 Fitness Trade‐Offs

To examine how trade‐offs contribute to adaptation, we modeled specific vital rates as the response variable of a three‐way interaction between the following predictors: a vital rate at a preceding stage of the life cycle, genotype, and growing environment. Specifically, we tested for three types of trade‐offs, (1) trade‐offs between flowering probability and plant size at the start of the same growing season, (2) trade‐offs between survival probability and plant size at the start of the same growing season; and (3) trade‐offs between winter survival probability and flowering probability in the previous growing season. For the trade‐off analyses involving plant size at the start of the first growing season we removed outlier individuals (low sites: 3 high (size > 1000 mm^2^) and 3 low (size > 2000 mm^2^) genotype individuals; high sites: 2 low (size > 5000 mm^2^) genotype individuals). Retaining these individuals in the analyses yields results that are qualitatively identical. We used generalized linear models with binomial error distribution to test for three‐way interactions, as well as two‐way interactions between vital rates and the genotype within the two environments. We determined the significance of the three‐way interactions using the Anova function in the R package (car) and two‐way interactions using likelihood ratio tests and computed pairwise contrasts between genotypes as well as confidence intervals of the trends using the R package emmeans (Lenth 2017).

#### 2.5.4 Elasticity Analyses and Stable Age Distributions

To elucidate the influence of individual life history traits (i.e., age‐specific survival and reproduction) on population growth of the genotypes growing in the low and high elevation environments, we extracted the elasticities of specific vital rates and stable age distributions from the MPM. The elasticities estimate the proportional sensitivities of a change in a specific vital rate to population growth rate, thus yielding their relative importance. As an outcome of trade‐offs between survival and reproduction, stable age distributions describe the proportion of the population that is found in each age class at equilibrium. Hence, the elasticities of genotypes growing in the alternative environment reflect estimates of the environmental effect on the importance of specific vital rates for population growth rates (Caswell 2001). We compared estimates at different stages of the life cycle by computing 95% bias corrected confidence intervals from 20,000 bootstrap replicates of the matrix models. To further describe the environmental effect on the elasticity values, we correlated the shift (i.e, difference in value when growing in a home vs. a foreign environment) and distance (i.e., difference in value between the two genotypes growing in their home environments) of the three populations representing each genotype. We refrained from assessing significance of the correlations because elasticities are non‐independent parameters derived from the population growth models.

## 3 Results

### 3.1 Evidence for Adaptation From Individual Vital Rates

**FIGURE 2:**
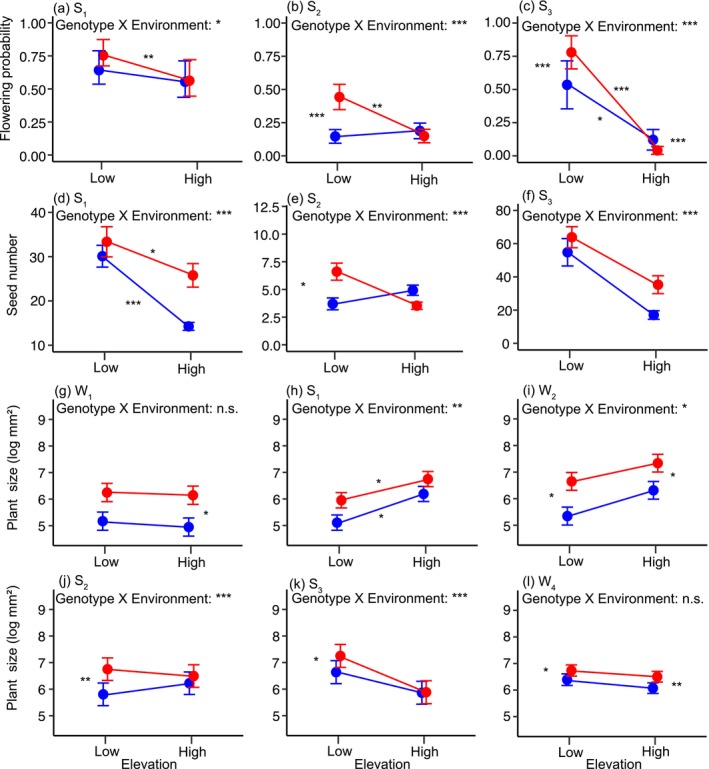
Reaction norms of elevational genotypes for reproduction (a–f) and plant size (g‐l) at subsequent stages of the life cycle. Symbols indicate mean estimate values inferred from mixed effect models and bars indicate 95% confidence intervals. Mean values are connected by reaction norms depicting the effect of the environment on each genotype. Red and blue colors denote the low and high genotypes, respectively. S_i_ and W_i_ denote summers and winters, respectively, of subsequent years. Significance of GxE interactions and contrasts consistent to the local vs. foreign and home vs. away criteria are reported (***p < 0.001, **p < 0.01, *p < 0.05).

We found significant GxE interactions for flowering probability in each year (Figure 2a–c, Table S5). The local genotype had significantly higher flowering probability at low elevation the second and third year, and at high elevation in the third year, thus fulfilling the local vs. foreign criterion. The low genotype also performed significantly better at home than away across the 3 years.

Reaction norms for seed number recapitulated patterns observed for flowering probability. Significant G × E interactions in each year (Figure 2d–f; Table S5) were associated with a fitness advantage of the local genotype at low elevation the second year. This genotype also showed home vs. away advantage throughout the experiment, albeit only statistically significant the first year.

Reaction norms for plant size varied substantially across the different stages of the plant life cycle (Figure 2g–l, Table S6). Significant GxE interactions were found at the start of each vegetation period (S_1_–S_3_), and over the second winter (W_2_). During the entire duration of the experiment, the low elevation genotype was consistently larger than the high elevation counterpart, even when growing at high elevation, though differences were not always statistically significant.

**FIGURE 3:**
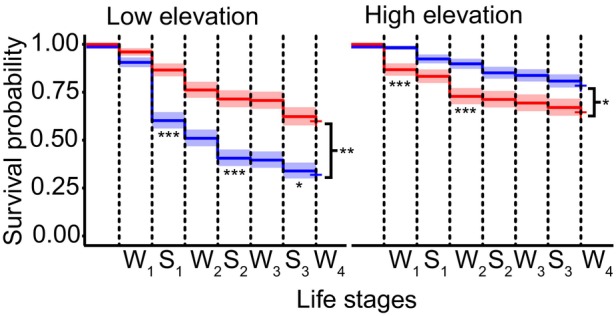
Survival of the elevational genotypes throughout the experiment at subsequent stages of the life cycle. Red and blue colors denote the low and high elevation genotypes, respectively. Survival curves of the two genotypes are inferred from cox proportional hazard models, with shaded areas representing 95% confidence intervals. S_i_ and W_i_ denote summers and winters, respectively, of subsequent years. Asterisks at the side of each plot indicate significant cumulative differences between the genotypes. Asterisks underneath the curves indicate significant differences of survival probability between genotypes at specific life stages, as assessed by generalized linear mixed effect models (***p < 0.001, **p < 0.01, *p < 0.05).

Cumulative survival was significantly higher in the local genotype in both environments (Figure 3; Table S7) with a stronger cumulative effect at low elevation. While the low elevation genotype showed similar cumulative survival at both elevations, the high elevation genotype performed significantly better in its home environment (Table S7). The most pronounced differences of the survival curves between genotypes were observed at alternative time points in the two environments. At low elevation, relative survival rates of the local genotype were significantly higher than the foreign genotype during the first two summer seasons (S_1_ and S_2_), with a particularly pronounced difference during the first (Figure 3; Table S8). In contrast, at high elevation, survival rates of the local genotype were significantly higher than the foreign genotype during the first and second winter (W_1_ and W_2_; Figure 3; Table S8).

### 3.2 Evidence for Adaptation From the Integrated Fitness Estimates

**FIGURE 4:**
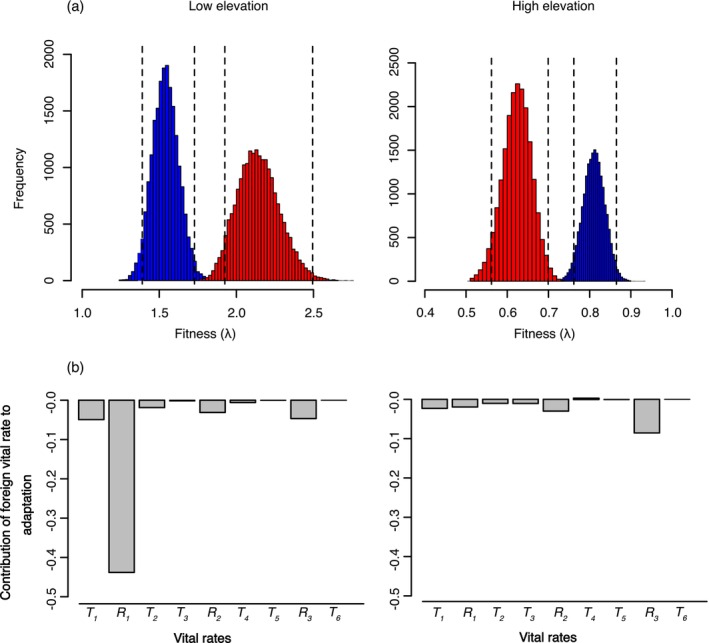
Integrative estimates of fitness. (a) Histograms representing population growth rate distributions based on 20,000 bootstrap replicates. Dotted lines indicate bias corrected 95% confidence intervals. Red and blue colors denote the low and high elevation genotypes, respectively. (b) LTRE showing the relative contribution to population growth of vital rates expressed as survival and reproduction (T_i_ and R_i_, respectively) of the foreign genotype at subsequent stages of the life cycle.

Comparisons of population growth rates inferred from MPM revealed a significant advantage of the local genotype in both the low and high elevation environment, with λ of the local genotype surpassing that of the foreign genotype by 39% and 31%, respectively (Figure 4; Table S9). LTRE analyses showed a negative impact of the foreign genotype on vital rates underlying population growth, predominantly driven by reproduction over survival (Figure 4; Table S9). Notably, differences in population growth were largely driven by reproduction in the first year in the low environment. At high elevation, the impact of individual vital rates was more evenly spread, with the strongest difference observed in the third year (R_3_).

### 3.3 Fitness Trade‐Offs

**FIGURE 5:**
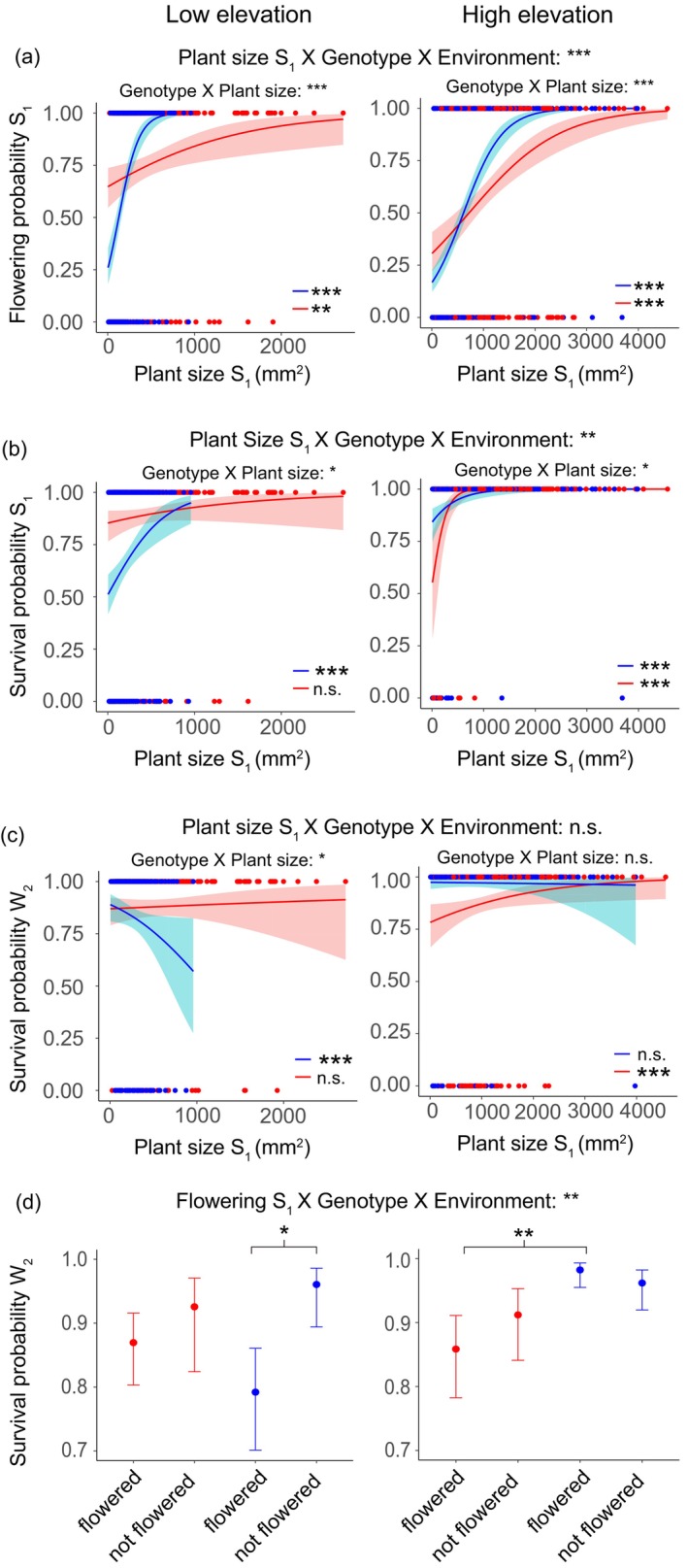
Fitness trade‐offs expressed by the genotypes. (a–c) The effect of size on flowering probability the first summer (S_1_), survival probability the first summer and second winter (W_2_). Lines indicate predicted relationships from generalized linear model regressions with 95% confidence intervals. Symbols indicate empirical values of plant size and flowering probability. Red and blue colors denote the low and high elevation genotypes, respectively. Significant relationships are indicated within each panel as asterisks (***p < 0.001, **p < 0.005, *p < 0.05). (d) The effect of flowering the first year on survival probability the second winter (W_2_). Symbols indicate mean estimate values inferred from generalized linear models and bars indicate 95% confidence intervals. Red and blue colors denote the low and high elevation genotypes, respectively. In all plots, for each vital rate used as response variable, significance of the three‐way interaction between the vital rate used as predictor, the genotype and the transplant elevation, as well as the two‐way interaction between the vital rate used as predictor and the genotype within the low and high elevation is reported (***p < 0.001, **p < 0.005, *p < 0.05). [Correction added on 12 November 2024, after first online publication: Figure 5A has been corrected to fix a rendering error.]

- *Size and flowering probability*. The flowering probability varied as function of a significant three‐way interaction among plant size at the start of summer, genotype and environment in the first and second year (Figure 5a; Table S10). Flowering probability consistently increased with increasing plant size, though with a stronger effect in the high compared to the low elevation genotype in both environments.
- *Size and survival*. Plant size generally showed a positive effect on survival throughout the experiment, with significant effects at multiple stages of the life cycle (Table S11). These effects were particularly relevant during the first year of flowering and subsequent winter. In the former season, we recovered a significant three‐way interaction, and larger size was generally associated with higher survival (Figure 5b; Table S11). However, the high elevation genotype showed contrasting effects on survival in the first summer (S_1_) and the following winter (W_2_), as larger plants suffered lower survival in the latter season (Figure 5c; Table S11).
- *Flowering probability and survival*. Survival varied as a function of a significant three‐way interaction among flowering probability, genotype and environment at the end of the first summer and the following winter. In the former season, flowering had a significant positive effect on survival of each genotype in each environment (Table S12). Conversely, in the latter it negatively affected the survival of the high genotype in the low elevation environment (Figure 5d; Table S12). Moreover, low elevation plants that flowered in the high elevation environment suffered higher mortality relative to the local flowering plants (Figure 5d; Table S12).

### 3.4 Elasticities and Stable Age Distributions

**FIGURE 6:**
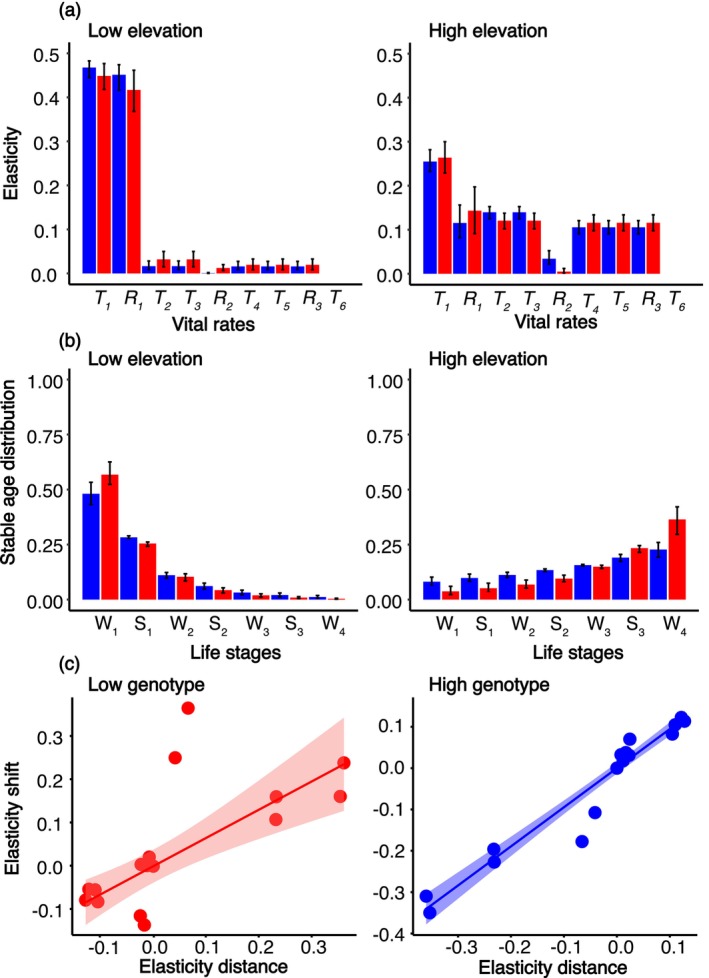
Elasticities and stable age distributions of the genotypes. (a) Influence of the specific vital rates of survival and reproduction (T_i_ and R_i_, respectively) on population growth rates. (b) Stable age distribution at subsequent summer and winter seasons (S_i_ and W_i_, respectively). In a–b, red and blue bars indicate estimates for the low and high genotypes, respectively. (c) Relationship between elasticity shift (i.e., difference in elasticity of age specific vital rates when growing at home vs. foreign environment) and elasticity distance (i.e., difference in elasticity of age specific vital rate between the two genotypes growing at the home elevation) expressed by the three focal populations (symbols) representative of each genotype growing at their respective home and away elevation. The shaded area indicates 95% confidence intervals of the trendline.

Elasticity values showed divergent patterns describing the influence of subsequent vital rates on population growth (Figure 6a; Table S13). Survival during the first winter (T_1_) and reproduction the first summer (R_1_) had by far the strongest influence at low elevation. In comparison, vital rates were more evenly spread at high elevation. Notably, elasticity values of both genotypes were similar within environments, with non‐significant differences at each stage of the life cycle. Similarly, stable age distributions differed substantially between environments, but showed similar trends between genotypes within environments. At low elevation, populations consisted primarily of young individuals, with a marked shift to adults at high elevation (Figure 6b; Table S14). Additionally, we observed correlations between elasticity shifts (i.e., difference in elasticity of age specific vital rates when growing a home vs. foreign environment) and distance (i.e., difference in elasticity of age specific vital rate between the two genotypes growing at the home elevation) for both genotypes (Figure 6c) in response to the high and low elevation environments.

## 4 Discussion

### 4.1 Elevational Adaptation in *D. carthusianorum*

Elevational gradients correspond to steep ecological gradients where spatially divergent natural selection acting on local populations has driven the evolution of adaptive traits in many plant species (e.g., Byars, Papst, and Hoffmann 2007; Kim, Donohue, and Jacquemyn 2013; Halbritter et al. 2018). Our multi‐year reciprocal transplant experiment conducted on young plants of *D. carthusianorum* revealed that populations originating from the lower and upper limits of the elevational distribution range in the central Alps are adapted to the contrasting environmental conditions that characterize these habitats. The most compelling evidence for elevational adaptation emerges from integrated fitness estimates that account for the contributions of individual vital rates over multiple years and reveal a strong advantage of the local over the foreign genotype in both low and high elevation environments. When this signal is decomposed into the contributions of individual vital rates, we find that both reproduction and survival contribute significantly to adaptation. While survival is commonly found to underly adaptation across plant species along elevational gradients, reproduction shows more inconsistent trends (Halbritter et al. 2018). Flowering probability and seed number, our two fitness components related to reproduction, showed multiple instances of significant GxE interaction underlying a fitness advantage of the local ecotype. These fitness components are not functionally correlated as they are linked to bolting and resource allocation during seed set, respectively (Angert and Schemske 2007; Hautier et al. 2009), and thus contribute independently to adaptation. The local genotype also consistently outperformed the foreign one in terms of survival. At low elevation, selection against the foreign genotype was most pronounced during summer, when plants are exposed to the high temperatures and seasonal drought (Figure S3) that characterize the climate of the colline elevational belt in the central Alps (Braun‐Blanquet 1961; Zumbrunnen et al. 2009). In contrast, the high elevation environment imposed particularly strong selection during winter, most likely because of the depletion of resources during the extended snow cover. Along with evidence of adaptation emerging from individual vital rates, our results show extensive variation in the signal of GxE interactions recovered across years. Yearly variation of environmental conditions may alter natural selection acting on populations, as shown in replicated experiments on annual or short‐lived species (e.g., Ågren and Schemske 2012; Oakley et al. 2023; Troth et al. 2018). In our experiment, year‐specific patterns of GxE interactions cannot be disentangled from annual climatic variation. However, MPM offer a straightforward way of accounting for these effects during the life cycle for inference of adaptation during one generation. Overall, our results indicate that spatially divergent natural selection associated with contrasting climatic conditions drove the evolution of populations that are adapted to their environment. Hence, we consider the low and high elevation genotypes as distinct ecotypes *sensu* Turesson (1922), and refer to them as such hereafter.

In our study, we set out to use plant size as a fitness proxy since biomass has been shown to share a positive relationship with fecundity and fitness in many plant species (Younginger et al. 2017). However, we found that plants originating from low elevation were consistently larger than plants from high elevation, regardless of the growing environment. Similar patterns across years indicate a strong genetic basis of this trait. This result is consistent with functional reconstructions of trait evolution in these populations (de Vries et al. 2024) as well as with observations of other plant species occurring along elevational gradients, where larger plants are commonly found at lower elevations (Körner 2003; Halbritter et al. 2018). We therefore consider size in *D. carthusianorum* as a phenotypic trait that likely diverged across elevation in response to natural selection (Cheplick 2020; Boyko et al. 2023; Halbritter et al. 2018; Körner 2003; Midolo, Wellstein, and Schwinning 2020).

### 4.2 Life History Divergence and Plant Growth

The allocation of resources to reproduction and survival is a fundamental mechanism that characterizes adaptive life history strategies. In our study, age specific advantages across vital rates strongly suggested that divergent life history traits between low and high elevation ecotypes evolved in response to natural selection. Results from LTREs revealed that the contribution to adaptation is driven by intense reproduction in the first year at low elevation, while yearly contributions are more homogeneous at high elevation. For both ecotypes, vital rates expressed in the foreign environment were associated with trade‐offs. For example, plants of the low elevation ecotype that flowered at high elevation in the first year suffered higher mortality the following winter than local plants. Concomitantly, high elevation plants flowering at low elevation suffered from reduced survival compared to non‐flowering plants. This trade‐off affecting high elevation plants in the foreign environment is likely a consequence of the altered plant growth under different conditions: warmer temperatures at low elevation caused increased plant growth and flowering already in the first year, but at the cost of reduced survival over the following winter.

Our complementary evidence on the contribution of life history traits to fitness throughout the early stages of the plant life cycle reveals that selection at low elevation favors the evolution of a life history strategy characterized by high investment in early reproduction mediated by the achievement of large plant size, whereas selection at high elevation favors self‐maintenance (de Vries et al. 2024). This is reflected in stable‐age distributions, with populations predominantly composed of young individuals with intense reproduction at low elevation, and a life cycle similar to that of short‐lived species. This inference is congruent with expectations for plants inhabiting high energy environments characterized by abundant resources and intense competition, where early reproduction and high seed number are advantageous and compensate for high juvenile mortality (von Arx, Edwards, and Dietz 2006; Kim and Donohue 2011; Laiolo and Obeso 2017; DeMarche, Anger, and Kay 2020). In contrast, populations at high elevation are primarily composed of older individuals. A life cycle characterized by reduced annual reproduction and allocation of resources to self‐maintenance constitutes a better strategy under a short growing season and limited resources (Childs, Metcalf, and Rees 2010; Johnston and Pickering 2004; Milla et al. 2009). The different life history strategies expressed by our ecotypes at contrasting elevations are in line with observed differences in species occurring along elevation gradients, where short lived species with high reproductive investment are common at lower elevations and long lived species with enhanced offspring quality and bet hedging strategies predominate at high elevation (Boyko et al. 2023; Laiolo and Obeso 2017). We cannot assess from our data how much these results are affected by yearly variation in the environments, as this would require temporal replication of our multi‐year experiment. However, the first reproductive season in our experiment was not affected by any noticeable anomaly in climatic conditions, and it is important to note that our inferences based on LTRE integrate across life stages. Thus, we infer that the observed differences in *D. carthusianorum* reflect alternative strategies that are adaptive under the contrasting environmental conditions that characterize low‐ and high‐elevation habitats.

### 4.3 Plasticity of Life History Traits

We observed a considerable shift in the elasticities of both ecotypes when grown in the foreign environment. This suggests that the environment has an important effect on contributions to population growth. This evidence further aligns with differences in life history traits observed between ecotypes, where population growth is primarily driven by the early stages of the life cycle at low elevation, and by the late ones at high elevation. In line with Ensing and Eckert (2019), we interpret these results as indicative of co‐gradient plasticity of life history traits. This plasticity likely contributes to the fitness of the foreign ecotype at low and high elevation but is not sufficient to match the fitness of the local ecotype. Plasticity of life history traits is often observed across plant species and may be driven by strong seasonal fluctuations in environmental conditions (Hassel, Pedersen, and Söderström 2005; Ghalambor et al. 2007; Ensing and Eckert 2019; Acasuso‐Rivero et al. 2019). It has been suggested that rapid shifts in selection caused by anthropogenic climate change cannot be matched by a sufficient evolutionary response in perennial species (Anderson and Song 2020; Anderson, Wadgymar, and Angert 2019). Adaptive plasticity, as observed here in *D. carthusianorum*, may mitigate the negative effects of climate‐induced selection and allow longer persistence under adverse conditions, thus supporting the evolution of an adaptive response in the long term (Ghalambor et al. 2007; Fox et al. 2019; Vinton et al. 2022; West‐Eberhard 2003).

## 5 Conclusions

Our multi‐year reciprocal transplant experiment reveals the key role of early life history traits in the evolution of elevational adaptation. By combining MPM and analyses of fitness trade‐offs, we find that elevational adaptation in *D. carthusianorum* is driven by selection mediated by key stressors such as summer drought at low elevation, and differences in resource availability linked to the length of the growing season at high elevation. We propose that future studies should dissect the role of individual selective agents using field experiments, including manipulations, such as snow removal or drought treatments (Bushey et al. 2023) to establish a link between natural selection and the genetic response of alpine plants. Our study highlights the utility of using demographic models to study the complementary contribution of vital rates to fitness in perennial plants. In this study, we examined the early life stages, which constitute a critical portion of the plant life cycle. However, we expect that due to the divergent life history strategies, even stronger evidence of adaptation would emerge if fitness were recorded over additional years when the fitness advantage of the strategy of the high elevation plants should increase. While the demographic models we applied have a long history in ecology, these approaches have only recently begun to be integrated in adaptation studies (e.g., DeMarche, Kay, and Angert 2016; Goebl et al. 2022; but see Waser and Price 1985 for an older example) and represent an exciting opportunity to identify drivers of ecotype formation (Wadgymar et al. 2022).

## Author Contributions

**Aksel Pålsson:** conceptualization (supporting), data curation (lead), formal analysis (lead), investigation (equal), methodology (equal), project administration (equal), resources (equal), visualization (equal), writing – original draft (equal), writing – review and editing (equal). **Ursina Walther:** investigation (supporting), resources (equal). **Simone Fior:** conceptualization (equal), investigation (equal), methodology (equal), project administration (equal), supervision (equal), writing – original draft (equal), writing – review and editing (equal). **Alex Widmer:** conceptualization (equal), funding acquisition (lead), investigation (equal), methodology (equal), project administration (equal), resources (lead), supervision (equal), validation (equal), writing – original draft (equal), writing – review and editing (equal).

## Conflicts of Interest

The authors declare no conflicts of interest.

## Supporting information


Data S1.

## Data Availability

The data from the reciprocal transplant experiment used in this study are deposited in the public repository Dryad and is available at: https://doi.org/10.5061/dryad.gxd2547sj. R code used for all analyses and data from the establishment experiment are available at the Github repository: https://github.com/akselpaalsson.
